# Supplementary material for: Structural basis for antibody recognition of vulnerable epitopes on Nipah virus F protein
Source: Nat Commun. 2023 Mar 17;14:1494. doi: 10.1038/s41467-023-36995-y (PMC10021056; doi:10.1038/s41467-023-36995-y)
Supplement: Supplementary file 1 — Supplementary Information [file 41467_2023_36995_MOESM1_ESM.pdf]

Title:

Structural basis for antibody recognition of vulnerable epitopes on Nipah virus F protein

Author List: Patrick O. Byrne[1], Brian E. Fisher[2], David R. Ambrozak[3], Elizabeth G. Blade[1], Yaroslav Tsybovsky[4], Barney S. Graham[2,5], Jason S. McLellan[1]*, Rebecca J. Loomis[2,6]*

Affiliations:

[1] Department of Molecular Biosciences, The University of Texas at Austin, Austin, Texas, USA 78712

[2] Viral Pathogenesis Laboratory, Vaccine Research Center, National Institute of Allergy and Infectious Diseases, National Institutes of Health, Bethesda, Maryland, USA 20892

[3] Immunology Laboratory, Vaccine Research Center, National Institute of Allergy and Infectious Diseases, National Institutes of Health, Bethesda, Maryland, USA 20892

[4] Vaccine Research Center Electron Microscopy Unit, Cancer Research Technology Program, Leidos Biomedical Research, Inc., Frederick National Laboratory for Cancer Research, Frederick, Maryland, USA 21701

[5] Current address : Morehouse School of Medicine, Atlanta, GA 30310

[6] Current address : GSK Global Health R&D Vaccines (GVGH), 53100 Siena

*Correspondence: rebeccajo.x.loomis@gsk.com (R.J.L.) and jmclellan@austin.utexas.edu (J.S.M.)

Supplementary Information

Supplementary Table 1. Nipah virus amino acid sequences

**Construct Name**	**Amino Acid Sequence**
>NiVF_prefusion	mysmqlascvtltlvllvnsQGILHYEKLSKIGLVKGVTRKYKIKSNPLTKDIVIKM<br>IPNVSNMSQCTGSVMENYKTRLNGILTPIKGALEIYKNNTHDCVGDVRLAGVCMAGV<br>AIGIATAAQITAGVALYEAMKNADNINKLKSSIESTNEAVVKLQETAEKTVYVFTAL<br>QDYINTNLVPTIDKIPCKQTELSLDLALSKYLSDLLFVFGPNLQDPVSNSMTIQAIS<br>QAFGGNYETLLRTLGYATEDFDDLLESDSITGQIIYVDLSSYYIIVRVYFPILTEIQ<br>QAYIQELLPVSFNNDNSEWISIVPNFILVRNTLISNIEIGFCLITKRSVICNQDYAT<br>PMTNNMRECLTGSTEKCPRELVVSSHVPRFALSNGVLFANCISVTCQCQTTGRAISQ<br>SGEQTLLMIDNTTCPTAVLGNVIISLGKYLGSVNYNSEGIAIGPPVFTDKVDISSQI<br>SSMNQSLQQSKDYIKEAQRLLDTVNPSLKLMKQIEDKIEEILSKIYHIENEIARIKK<br>LIGEAPGGLVPRGSHHHHHHSAWSHPQFEK-
>NiVF_postfusion	mysmqlascvtltlvllvnsQGILHYEKLSKIGLVKGVTRKYKIKSNPLTKDIVIKM<br>IPNVSNMSQCTGSVMENYKTRLNGILTPIKGALEIYKNGGSGVAIGIATAAQITAGV<br>ALYEAMKNADNINKLKSSIESTNEAVVKLQETAEKTVYVLTALQDYINTNLVPTIDK<br>ISCKQTELSLDLALSKYLSDLLFVFGPNLQDPVSNSMTIQAISQAFGGNYETLLRTL<br>GYATEDFDDLLESDSITGQIIYVDLSSYYIIVRVYFPILTEIQQAYIQELLPVSFNN<br>DNSEWISIVPNFILVRNTLISNIEIGFCLITKRSVICNQDYATPMTNNMRECLTGST<br>EKCPRELVVSSHVPRFALSNGVLFANCISVTCQCQTTGRAISQSGEQTLLMIDNTTC<br>PTAVLGNVIISLGKYLGSVNYNSEGIAIGPPVFTDKVDISSQISSMNQSLQQSKDYI<br>KEAQRLLDTVNPSLKLMKQIEDKIEEILSKIYHIENEIARIKKLIGEAPGGLVPRGS<br>HHHHHHSAWSHPQFEK-

Supplementary Table 2. Antibody VH/VL Amino Acid Sequences

**Antibody Name**	**Amino Acid Sequence**
>4H3_heavy_chain	QIQLVQSGPELKKPGETVKISCKASGYTFRNYGVNWVKQGPGKDLKWMGWINTLNGEPTYADDFKRRFAFSLETSATTAFLQINNLKNEDTATYFCARTFYDGYYYAMDYWGQGTSVTVSA
>4H3_light_chain	ENVLTQSPTIMAASLGQKVTMTCSANSSVSSSYLHWYHQKSGASPKPLIHRTSNLASGVPARFIGSGSGTSFSLTISSVEAEDDATYYCQQWSGYPFITFGSGTKLEIK
>2D3_heavy_chain	QVQLKQSGPGLVAPSQSLSITCTVSGFSLTSYALSWVRQPPGKGLEWLGVIWVGGVTNYNSALKSRLTISKDNSKSQVFLKMNSLQSEDTARYYCASRHLSTGAMDYWGQGTSVTVSA
>2D3_light_chain	DVLMTQSPLSLPVSLGDQASISCRSSQSIVHSNGDTYFEWYLQKPGQSPKLLIYKVSNRFSGVPNRFSGSGSGTDFTLKISRVEAEDLGVYYCFQGSYVPYTFGGGTKLEIK
>1H8_heavy_chain	EVQLQQSGPELVKPGASVKISCKASGYSFTGYTMNWVKQSHGKNLEWIGLINPFIGGTRYNQKFKGKATLTVDKSSRTAYMELLSLTSEDSAVYYCAREADYDWYFDVWGAGTTVTVSS
>1H8_light_chain	DIQMTQTTSSLSASLGDRVTISCRASQDISNYLHWYQQKPDGTVNLLIFYTSRLHSGVPSRFSGSGSGTDYSLTISNLEQEDIATYFCQQGNTLPRTFGGGTKLEIK
>1A9_heavy_chain	QVQLQQSGAELVRPGTSVKISCKASGYTFTNYWLGWVKQRPGHGLEWIGDIYRGGGYTNYNEKFKGKATLTADTSSSTAYMQLSSLTSEDSAVYFCATRDGYFDYWGQGTTLTVSS
>1A9_light_chain	DIQMTQSSSSFSVSLGDRTTITCKASEDIYNRLAWFQQKPGNAPRLLISGATSLETGVPSRFSGSGSGKDYTLSITSLQTEDVATYYCQQYWSSPWTFGGGTKLEIK
>1H1_heavy_chain	QVQLQQSGAELMRPGASMKISCKATGYTFSSYWIDWVKQRPGHGLEWIGEILPGSGDTNYNENFKGKAAFTADTSSNTAYMQLTSLTSEDSAVFYCARGGRYHGQGFFDYWGQGTTLTVSS
>1H1_light_chain	DIQMTQSPASLSASVGETVTITCRPSENVHIYLAWYQQKQGKSPQLLVYNAKTLADGVPSRFSGSASGTQFSLKINSLQPEDFGSYYCQHFWSIPYTFGGGPS
>2B12_heavy_chain	EVQLVESGGGLVQPKGSLKLSCAASGFIFNTYAMHWVRQAPGKGLEWVARIRSKSSNYATYYADSVKDRFTISRDDSQSMLYLQMNNLKTEDTAMYYCVREGGYYFDYWGQGTTLTFSA
>2B12_light_chain	DIVMSQSPSSLAVSVGEKVTMSCKSSQSLLHVSNQKNYLAWYQQKPGQSPKLLIYWASTRESGVPDRFTGSGSGTDFTLTISSVKAEDLAVYYCQQYYSYPWPFGGGTKLKSN
>1F2_heavy_chain	EVQLQQSGPELVKPGVSLKISCKASGYSFTDYTMNWVKQSHGKNLEWIGLINPYIGGTRYNQKFKGKATLTVDKSSSTAYLELLSLTSEDSAVYYCARFSGSSNRAMDYWGQGTSVTVSS
>1F2_light_chain	DIQMTQSPASLSASVGETVTITCGASENIYGALNWYQRKQGKSPQLLIYGATNLADGMSSRFSGSGSARQYSLKISSLHPDDVATYYCQNVLSIPWTFGGGTKLEIK
>1F3_heavy_chain	EVQLQESGPSLVKPSQTLSLTCSVTGDSITSGYWNWIRKFPGNSLDYMGYIRYSGSTYYNPSLKSRISITRDTSKNQYYLQLNSVTTEDTATYYCARSGGLLGYAMDYWGQGTSVTVSS
>1F3_light_chain	DIQMTQITSSLSASLGDRVTISCRASQDIGNYLNWYQQKPDGTVKLLIYYTSRLHSGVPSRFSGSGSGTDYNLTITNLEQEDIATYFCQQGNTLPRTFGGGTKLEIK
>4B8_heavy_chain	EVQLQQSGPELVKPGASMKISCKASGYSFTGYTMNWVKQSHGKNLEWIGLINPYIGGTRYNQKFKDKATLTVDKSSSTAYMELLSLTSEDSAVYYCAREADYDWYFDVWGAGTTVTVSS
>4B8_light_chain	DIQMTQTTSSLSASLGDRVTISCRASQDISNYLNWYQQKPDGTVKLLIYYTSRLHSGVPSRFSGSGSGTDYSLTISNLDQEDIATYFCQQGNTLPRTFGGGTKLEIK
>4F6_heavy_chain	QVQLQQSGAELVKPGASVKLSCKASGYTFTSNWMHWVKQRPGRGLEWIGRIDPNSGGTKYNEKFKSKATLTVDKPSSTAYMQLSSLTSEDSAVYYCARDGHYAYFDVWAPGTTVTVSS
>4F6_light_chain	DIVMTQSQKFMSTSVGDRVSVTCKASQNVGTTVAWYQQKPGQSPNALIYSASYRYTGVPDRFTGSGSGTDFTLTISNVQSEDLAEYFCQQYNRYPWTFGGGTKLEIK

Supplementary Table 3. Cryo-EM Data Collection, Reconstruction and Model Validation

**EM DATA COLLECTION**						
Microscope (FEI)	Titan Krios	Titan Krios	Titan Krios	Titan Krios	Talos Glacios	Titan Krios
Voltage (kV)	300	300	300	300	200	300
Detector	Gatan K3	Gatan K3	Gatan K3	Gatan K3	Falcon 4	Gatan K3
Pixel size (Å/pix)	1.1	0.66	1.1	0.81	0.94	0.66
Exposure rate (e^-/pix/s)	8	8	8	10	6	8
Frames per exposure	80	80	80	40	60	80
Exposure (e^-/Å^2)	80	80	80	70	40	80
Defocus range (µm)	1.5-2.5	1.5-2.5	1.5-2.5	1.0-2.5	1.0-2.0	1.5-2.5
Tilt angle (degrees,°)	30	0	0	30	30	30
Micrographs collected	1,008	3,717	3,168	1,836	1,602	6,498
Micrographs used	962	3,390	2,825	1,449	748	5,098
Particles extracted	1,074,760	2,342,042	4,546,609	772,998	287,845	2,869,920
Automation software	SerialEM	SerialEM	SerialEM	SerialEM	SerialEM	SerialEM
**Complex Composition**	**NiV F + Fab 4H3**	**NiV F + Fab 2D3**	**NiV F + Fab 1H8**	**NiV F + Fab 1A9**	**NiV F + Fab 1H1**	**NiV F + Fab 2B12**
**FINAL 3D RECONSTRUCTION STATISTICS**						
Particles	291,096	104,409	305,954	236,894	96,726	694,547
Symmetry	C3	C1	C3	C3	C3	C3
Map sharpening B-factor	138	133	116	101	100	137
Resolution at FSC...						
Unmasked: 0.5 (Å)	4.2	7.6	4.3	3.5	4.1	3.5
Masked: 0.5 (Å)	3.1	3.2	2.9	3.1	3.4	2.6
Unmasked: 0.143 (Å)	3.5	4.0	3.5	3.2	3.6	3.1
Masked: 0.143 (Å)	2.8	2.8	2.5	2.8	3.0	2.4
**MODEL REFINEMENT AND VALIDATION STATISTICS**						
Composition						
Amino Acids (#)	2010	1556	2016	1983	2025	2016
Ligands (Type: #)	NAG: 12	NAG: 14	NAG: 12	NAG: 12	NAG: 3	NAG: 12
RMSD Bonds						
Length [Å] (# > 4s)	0.004 (0)	0.004 (0)	0.003 (0)	0.004 (0)	0.003 (0)	0.002 (0)
Angles [°] (# > 4s)	0.94 (0)	0.95 (0)	0.63 (0)	0.96 (0)	0.56 (0)	0.55 (0)
Ramachandran plot						
Outliers (%)	0.00	0.00	0.00	0.00	0.00	0.00
Allowed (%)	2.42	2.66	3.87	1.79	1.89	1.71
Favored (%)	97.58	97.34	96.13	98.21	98.11	98.29
Rotamer outliers (%)	1.02	0.51	0.96	0.06	0.68	1.13
C-β outliers (%)	0.00	0.00	0.00	0.00	0.00	0.00
CaBLAM outliers (%)	0.82	0.59	0.41	1.09	1.01	0.71
CC (mask)	0.83	0.84	0.85	0.79	0.84	0.89
MolProbity score	1.11	1.15	1.22	1.24	1.20	0.97
Clash score	2.36	2.37	1.94	4.68	4.17	1.74
**Complex Composition**	**NiV F + Fab 4H3**	**NiV F + Fab 2D3**	**NiV F + Fab 1H8**	**NiV F + Fab 1A9**	**NiV F + Fab 1H1**	**NiV F + Fab 2B12**
**EMDB ID**	26652	26658	26659	26668	26660	26662
**PDB ID**	7UOP	7UP9	7UPA	7UPK	7UPB	7UPD

Supplementary Table 4. Mouse BCR and Sequencing Primers

1st round IgH		1st round IgK	
VH1 Ext	agRtYcagctgcaRcagtct	Vkappa1 Ext	tgatgacccaRactccact
VH1-2 Ext	aggtccaactgcagcagcc	Vkappa2 Ext	Gcttgtgctctggatccc
VH2 Ext	tctgcctggtgacWttccca	Vkappa3 Ext	Ctgctgctctgggttcc
VH3 Ext	gtgcagcttcaggagtcag	Vkappa4 Ext	cagcttcctgctaatcagtg
VH4 Ext	gaggtgaagcttctcgagtc	Vkappa5 Ext	ctcagatccttggacttHtg
VH5 Ext	gaagtgaagctggtggagtc	Vkappa6 Ext	tggagtcacagacYcagg
VH6 Ext	atgKacttgggactgaRctgt	Vkappa7 Ext	Tggagtttcagacccagg
VH7 Ext	cagtgtgaggtgaagctggt	Vkappa8 Ext	ctgctMtgggtatctggt
VH8 Ext	ccaggttactctgaaagagtc	Vkappa9 Ext	cWtcttgttgctctggtttc
VH9 Ext	tgtggaccttgctattcctga	Vkappa10 Ext	Gatgtcctctgctcagttc
VH10 Ext	tgttggggctgaagtgggttt	Vkappa11 Ext	Cctgctgagttccttggg
VH11 Ext	atggagtgggaactgagctta	Vkappa12 Ext	Ctgctgctgtggcttaca
VH12 Ext	agcttcaggagtcaggacc	Vkappa13 Ext	Ccttctcaacttctgctct
VH13 Ext	caggtgcagcttgtagagac	Vkappa14 Ext	agggcccYtgctcagttt
VH14 Ext	atgcagctgggtcatcttctt	Vkappa15 Ext	Atgagggtccttgctgag
VH15 Ext	gactggatttggatcacKctc	Vkappa16 Ext	Gaggttccaggttcaggt
VH16 Ext	tggagtttggacttagttggg	Vkappa17 Ext	ccatgaccatgYtctcact
Cgamma Ext	agggaaataRcccttgaccag	Vkappa18 Ext	Atggaaactccagcttcattt
Cgamma-2 Ext	agggaagtagcctttgacaag	Vkappa19 Ext	Atgagaccgtctattcagtt
		Ckappa Ext	Gcacctccagatgttaactg
2nd round IgH		2nd round IgK	
VH1 Int	gaRgatRtcctgYaaggcttc	Vkappa1 Int	cctgtcagtcttggagatca
VH1-2 Int	aRgBtgtcctgcaagRcttc	Vkappa1-2 Int	Tttgtcggttaccattggacaa
VH2 Int	tgcagctgaagSagtcagga	Vkappa2 Int	SRgatattgtgatgacgcagg
VH3 Int	aaccttctcagWcactgtcc	Vkappa3 Int	Attgtgctgacccaatctcc
VH4 Int	ggaggtggcctggtgcag	Vkappa4 Int	aWtgtKctcacccagtctcc
VH5 Int	agcctggagggtccctgaa	Vkappa5 Int	Gtctccagccaccctgtc
VH6 Int	gaggagtctggaggaggctt	Vkappa6 Int	tgatgacccagtctcMcaaat
VH7 Int	tctggaggaggcttggtaca	Vkappa7 Int	gcctgtgcagacattgtgat
VH8 Int	ctgggatattgcagccctcc	Vkappa8 Int	cctgtggggacattgtgatg
VH9 Int	acagatccagttggtgcagt	Vkappa9 Int	acatccRgatgacYcagtct
VH10 Int	aggtgtgcattgtgaggtgc	Vkappa10 Int	ccagatgtgatatccagatg
VH11 Int	gaagtgcagctgttggagac	Vkappa11 Int	gccagatgtgatgtYcaaatg
VH12 Int	cctggtgaaaccctcacag	Vkappa12 Int	Atccagatgactcagtctcc
VH13 Int	aggcttggtgaggcctgga	Vkappa13 Int	cctgatatgtgacatccRVat
VH14 Int	gaggttcagctgcagcagt	Vkappa14 Int	Magatgacccagtctccatc
VH15 Int	caatcccaggttcacctacaa	Vkappa15 Int	tgagatgtgacatccagatga
VH16 Int	gtgaggtgcagctggtgga	Vkappa16 Int	ccagtgtgatgtccagataac
Cgamma Int	ggccagtggatagacHgatg	Vkappa17 Int	acaactgtgacccagtctcc
Cgamma-2 Int	cagggaccaagggatagaca	Vkappa18 Int	acacaggctccagcttctct
		Vkappa19 Int	gtgctcagtgtgacatccag
		Ckappa Int	gatggtgggaagatggatac

Chain	Sequencing Primers		
IgH-1	Cgamma Int		ggccagtggatagacHgatg
IgH-2	Cgamma-2 Int		cagggaccaagggatagaca
IgK	Ckappa Int		gatggtgggaagatggatac
IgL-1	Clambda Int		gaaacacggtgagWgtggg
IgL-2	Clambda-2 Int		Gaaacagggtgactgatgg

Supplementary Figure 1. Probe Development, Antibody Isolation and Characterization

A

Detector	Antibody	Color	Clone	Marker
B515	IgG1	FITC	A85-1	Plasma B cell
B515	IgG2	FITC	R2-40	Plasma B cell
B515	IgG3	FITC	R40-82	Plasma B cell
V450	B220	BV421	RA3-6B2	(CD45R) All stages of B cells
V510	F4/80	BV510	BM8	Dump; Macrophages and DC
V510	CD4	BV510	GK1.5	Dump; CD4 T cells
V510	Gr-1	BV510	R86-8C5	Dump; (Ly6G/Ly6C) Monocytes and Macrophages
V510	CD8	BV510	53-6.7	Dump; CD8 T cells
G780	IgM	PeCy7	1B4B1	Mature B cells
V510	Aqua	BV510		Viability
R670	PreF	APC		Probe

C

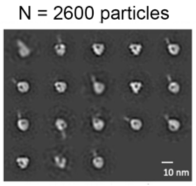

D

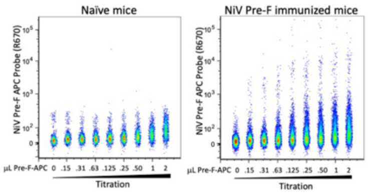

B

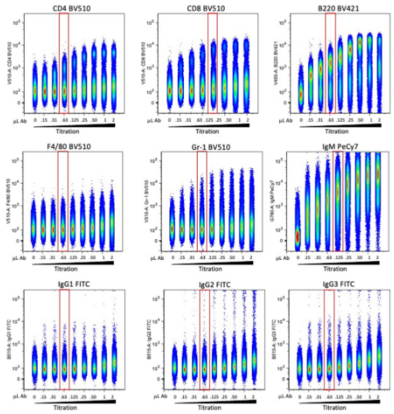

E

Pre-F Sorted H-L Pairs					
Pre-F Specific Binders			Pre-F and Post-F Specific Binders		
Antibody	Pre-F Response (nm)	Post-F Response (nm)	Antibody	Pre-F Response (nm)	Post-F Response (nm)
1H8*	2.6174	0.0288	4F4	2.4147	1.2131
2B12*	2.5568	0.0187	4D5	2.0014	1.5804
3D4	2.4555	-0.0009	3G2	1.8998	1.8179
2D3*	2.4212	0.077	1A6	1.8363	1.5647
3A4	2.2424	0.0034	3G11	1.8129	1.7384
4H3*	2.2243	0.0436	1H5	1.7767	1.608
3H11	2.1154	0.0491	3H3	1.4733	1.6754
4D8	2.1036	0.1365	4E11	1.425	1.5761
4F6*	1.8162	-0.0326	4A3	1.3428	1.5089
2D4	1.7652	0.0281	1H1*	1.2674	1.6275
1F2*	1.6636	-0.1369	3E1	0.954	1.0295
4B8*	1.5458	-0.0861	1G1	0.6603	0.8423
2C2	1.4774	-0.0898	3H6	0.5429	0.672
1A9*	1.4759	-0.0953	1A10	0.0559	0.1192
4C5	1.4345	-0.1043			
3C3	1.4152	-0.0929			
2D5	1.1373	-0.2038			
1F3*	1.0243	-0.1482			
4E1	1.004	-0.195			
4C7	0.2099	-0.194			
3B11	0.1352	-0.02			

F

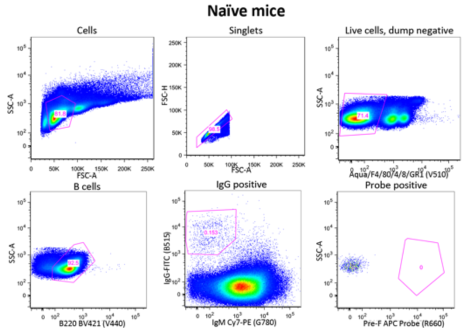

G

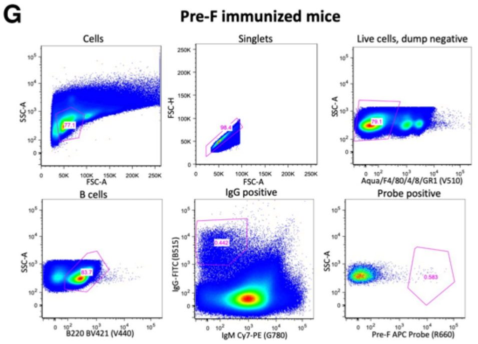

H

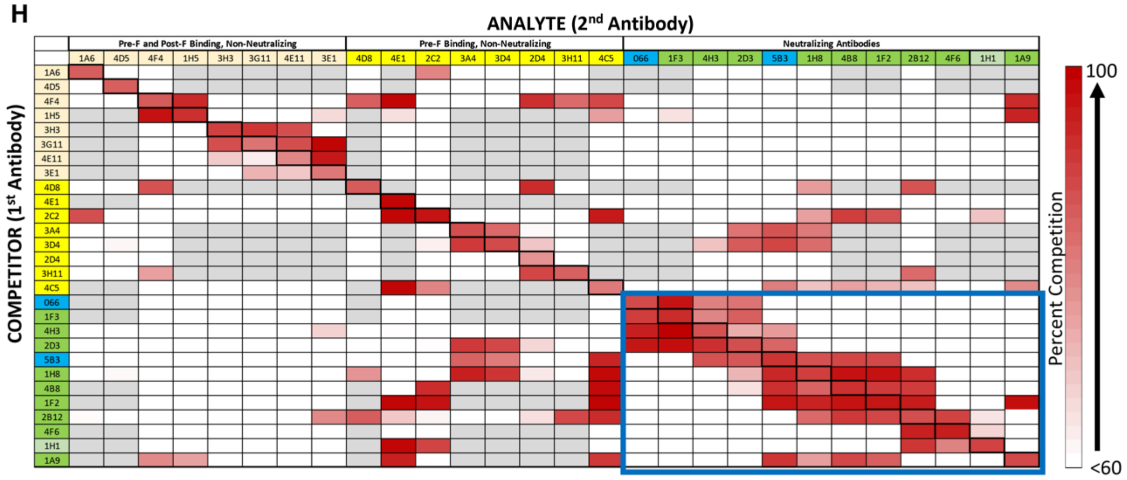

Supplementary Figure 1. Probe development, antibody isolation and characterization. (A) Final antibody panel used for B-cell sort. (B) Titration of antibodies used for the sort panel. (C) Negative-stain EM of biotinylated NiV pre-F probe protein. The total number of particles (N = 2600) is shown above the

2D class averages. (D) Titration of conjugated pre-F-APC probe on naïve mice or pre-F immunized mice splenocytes to determine optimal amount to use in B-cell sort FACS. (E) Biolayer interferometry specificity binding for expressed monoclonal antibodies. Asterisks indicate antibodies that were shown to be neutralizing via pseudovirus neutralization assay. Data is representative of two independent experiments. (F, G) Gating strategy for isolation of antigen-specific memory B cells by fluorescence activated cell sorting (FACS). The splenocytes were gated for antigen-specific memory B cells, specifically live IgG+ B cells which were also positive for the NIV prefusion F-APC probe (live, B220+, CD4-/CD8-/F4/80-/Gr-1-, IgM-, IgG+ and NiV prefusion F+). Cells index sorted 8 96-well plates using a FACS Aria II (BD Biosciences) interfaced with FacsDiva software (BD Biosciences). Flow cytometry analyses was performed using FlowJo software (Tree Star, Inc.). (H) Competition binding experiment, as measured by biolayer interferometry. Competitor antibodies are listed on the left by row, analyte antibodies are listed on the top by column. The percent competition is indicated by intensity of shading, from white (<60%) to red (100%). Antibodies are colored according to their binding specificities and neutralization characteristics, indicated in headings above antibody name on the top.

Supplementary Figure 2. Binding of Fabs to prefusion NiV F by biolayer interferometry

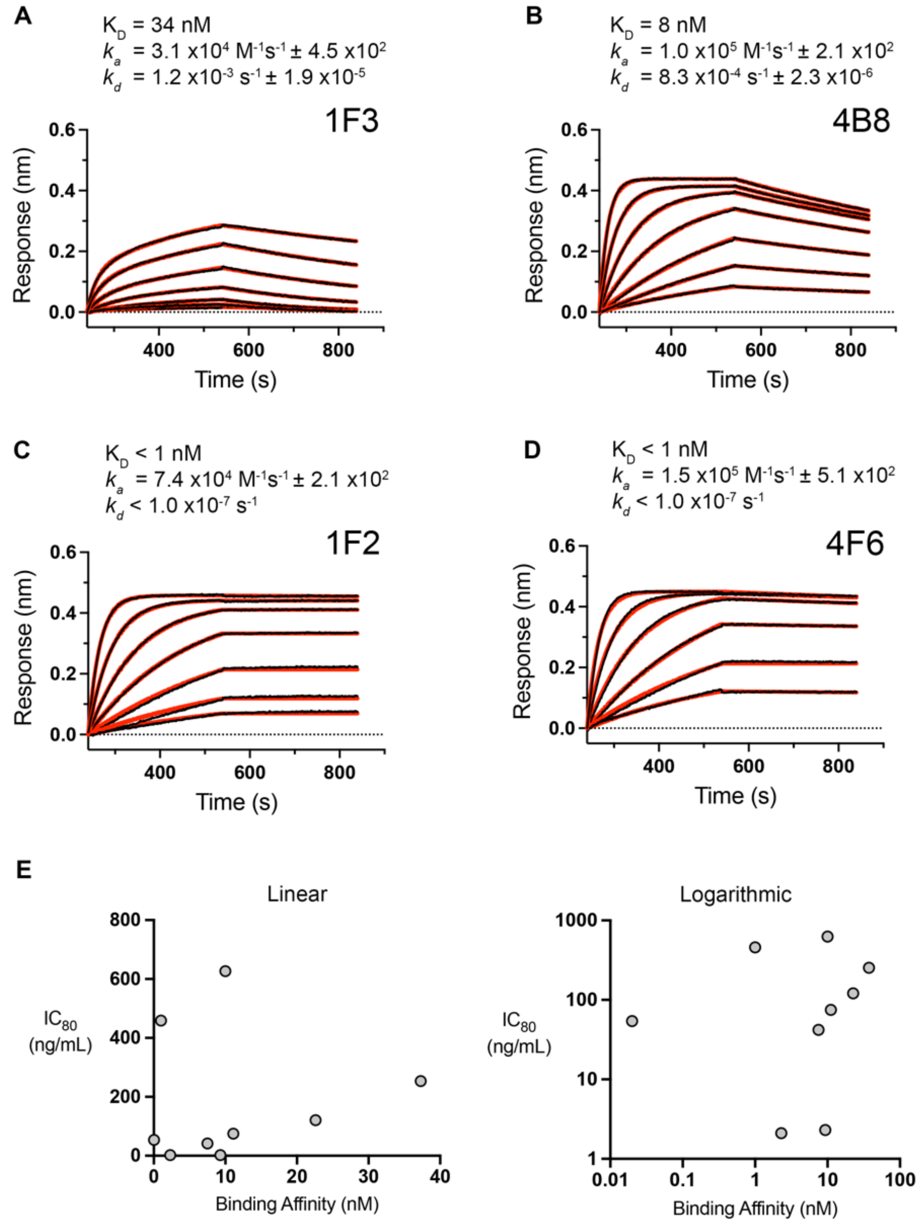

Supplementary Figure 2. Binding of Fabs to prefusion NiV F by biolayer interferometry. (A-D) Binding of Fabs to streptavidin biosensor tips functionalized with strep-tagged NiV prefusion F protein. The y-axis shows response units in nanometers, the x-axis shows time in seconds. Antibody identities are indicated at the top right of each graph. The raw sensorgrams are shown in black, and the lines of best fit to a 1:1 binding model are shown in red. Best fit values for the dissociation constant (K_D) are indicated within each graph. Data are representative of two biological replicates and two technical replicates. (E) Plot of neutralization potency (IC_{80}) as a function of Fab affinity for NiV F. Data correspond to the values listed in Table 1 in the main text.

Supplementary Figure 3. Cryo-EM Processing: Prefusion NiV F in complex with Fab 4H3

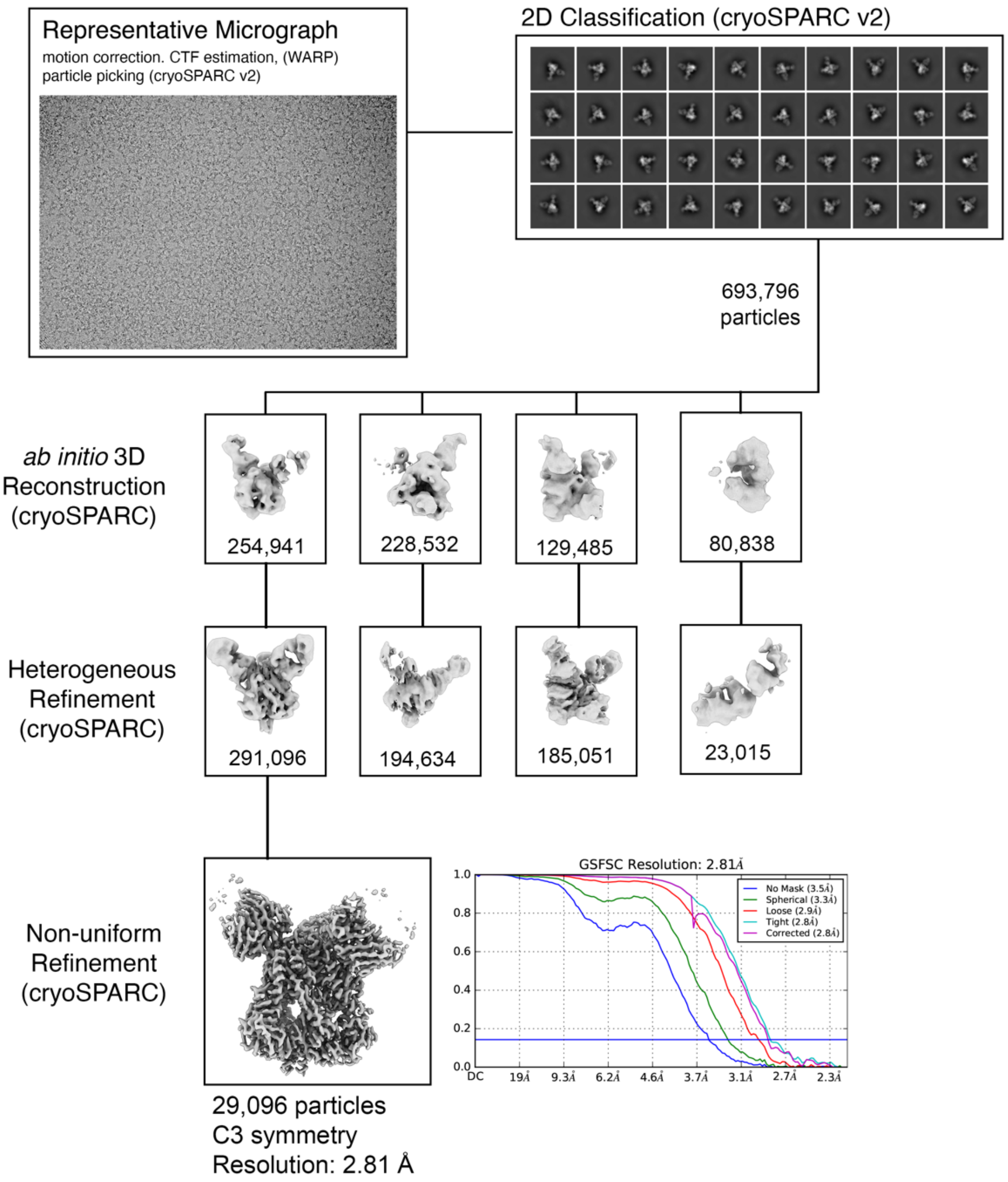

Supplementary Figures 3-8. Cryo-EM processing. Prefusion NiV F alone and in complex with Fabs 4H3, 2D3, 1H8, 1A9, 1H1, 2B12. Each supplementary figure shows a representative motion-corrected micrograph, 2D class averages, and volumes from 3D reconstruction and refinement. Particle numbers and software programs are listed beside each step. High-resolution 3D reconstructions are shown at the bottom of each page, along with their symmetry and gold standard Fourier shell correlation resolutions.

Supplementary Figure 4. Cryo-EM Processing: Prefusion NiV F alone and in complex with Fab 2D3

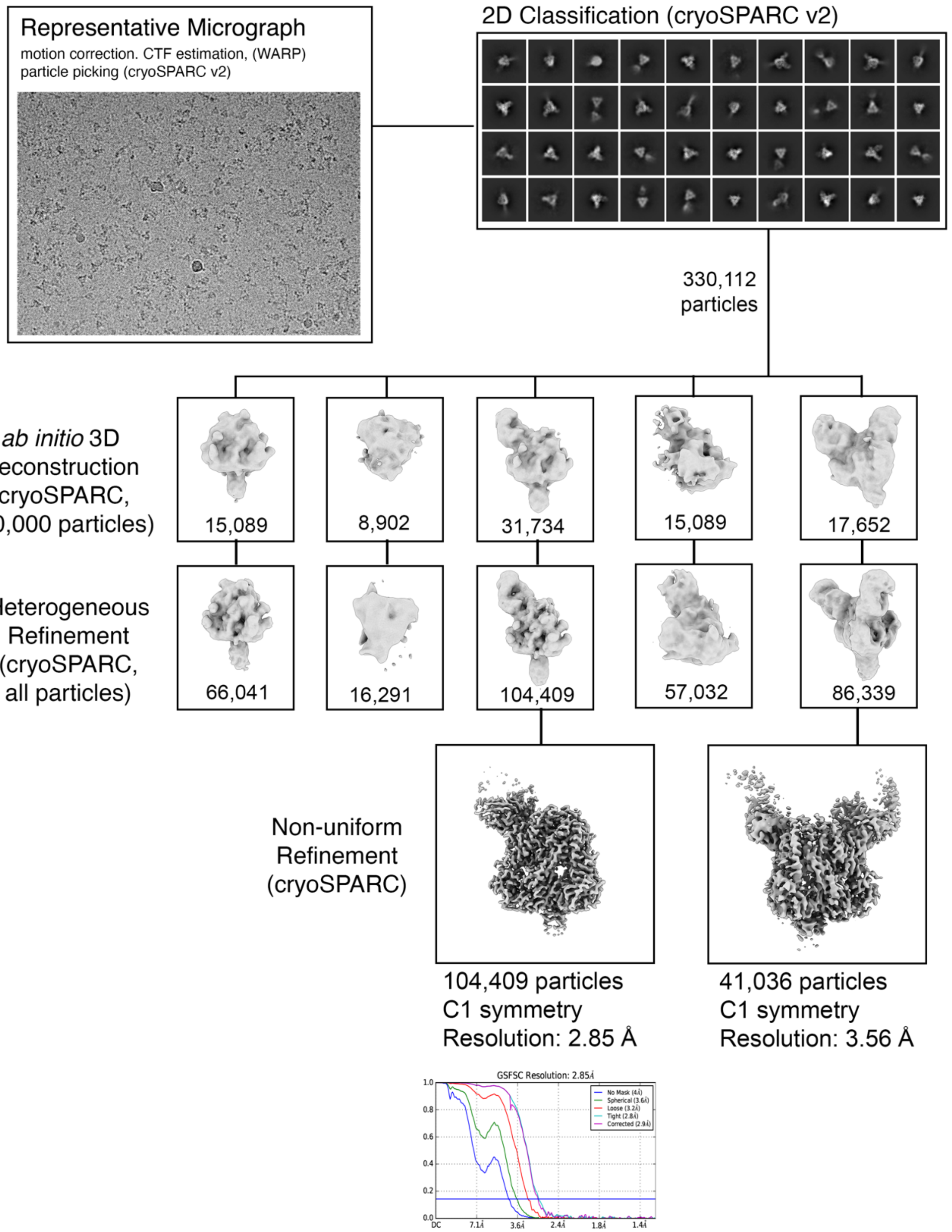

Supplementary Figures 3-8. Cryo-EM processing. Prefusion NiV F alone and in complex with Fabs 4H3, 2D3, 1H8, 1A9, 1H1, 2B12. Each supplementary figure shows a representative motion-corrected micrograph, 2D class averages, and volumes from 3D reconstruction and refinement. Particle numbers and software programs are listed beside each step. High-resolution 3D reconstructions are shown at the bottom of each page, along with their symmetry and gold standard Fourier shell correlation resolutions.

Supplementary Figure 5. Cryo-EM Processing: Prefusion NiV F in complex with Fab 1H8

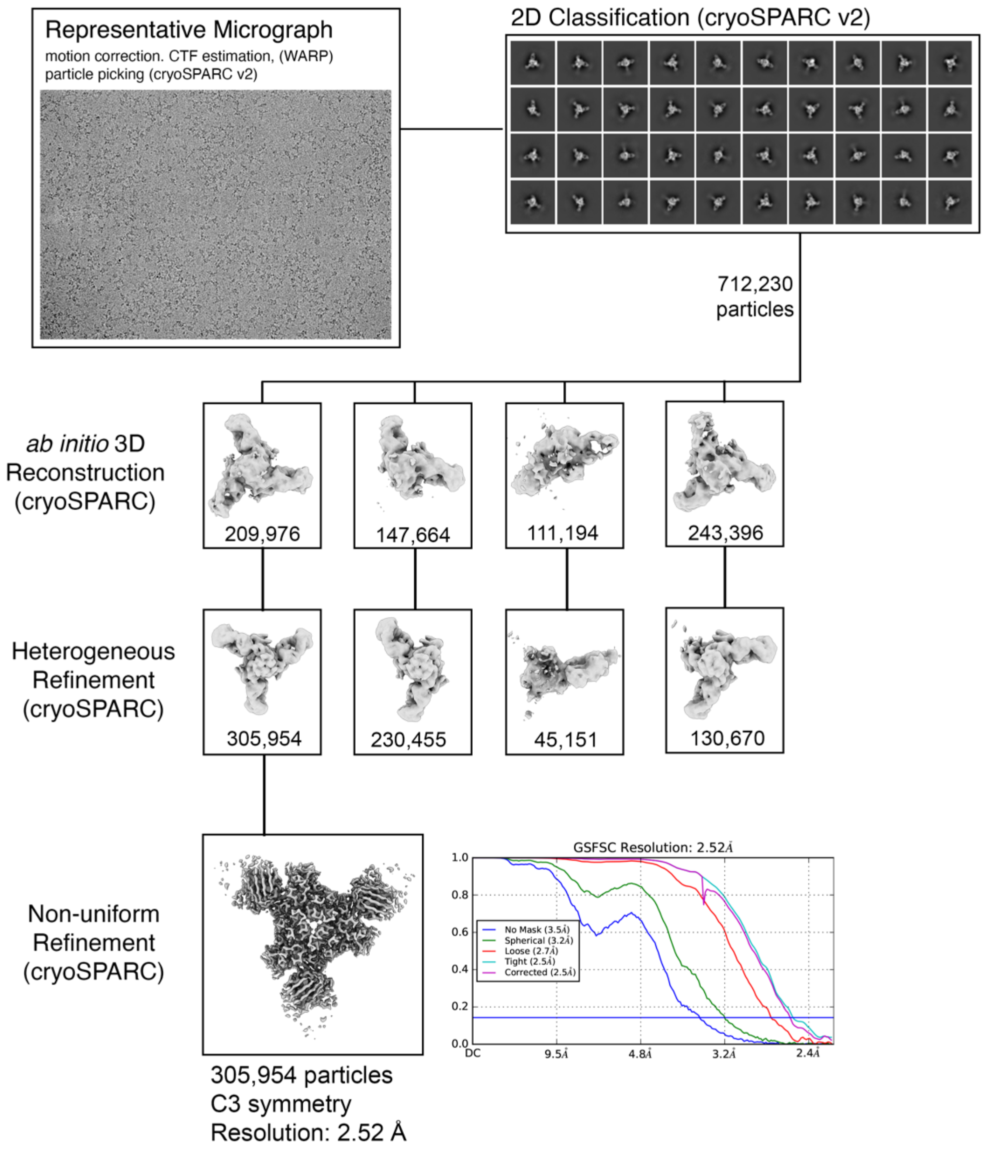

Supplementary Figures 3-8. Cryo-EM processing. Prefusion NiV F alone and in complex with Fabs 4H3, 2D3, 1H8, 1A9, 1H1, 2B12. Each supplementary figure shows a representative motion-corrected micrograph, 2D class averages, and volumes from 3D reconstruction and refinement. Particle numbers and software programs are listed beside each step. High-resolution 3D reconstructions are shown at the bottom of each page, along with their symmetry and gold standard Fourier shell correlation resolutions.

Supplementary Figure 6. Cryo-EM Processing: Prefusion NiV F in complex with Fab 1A9

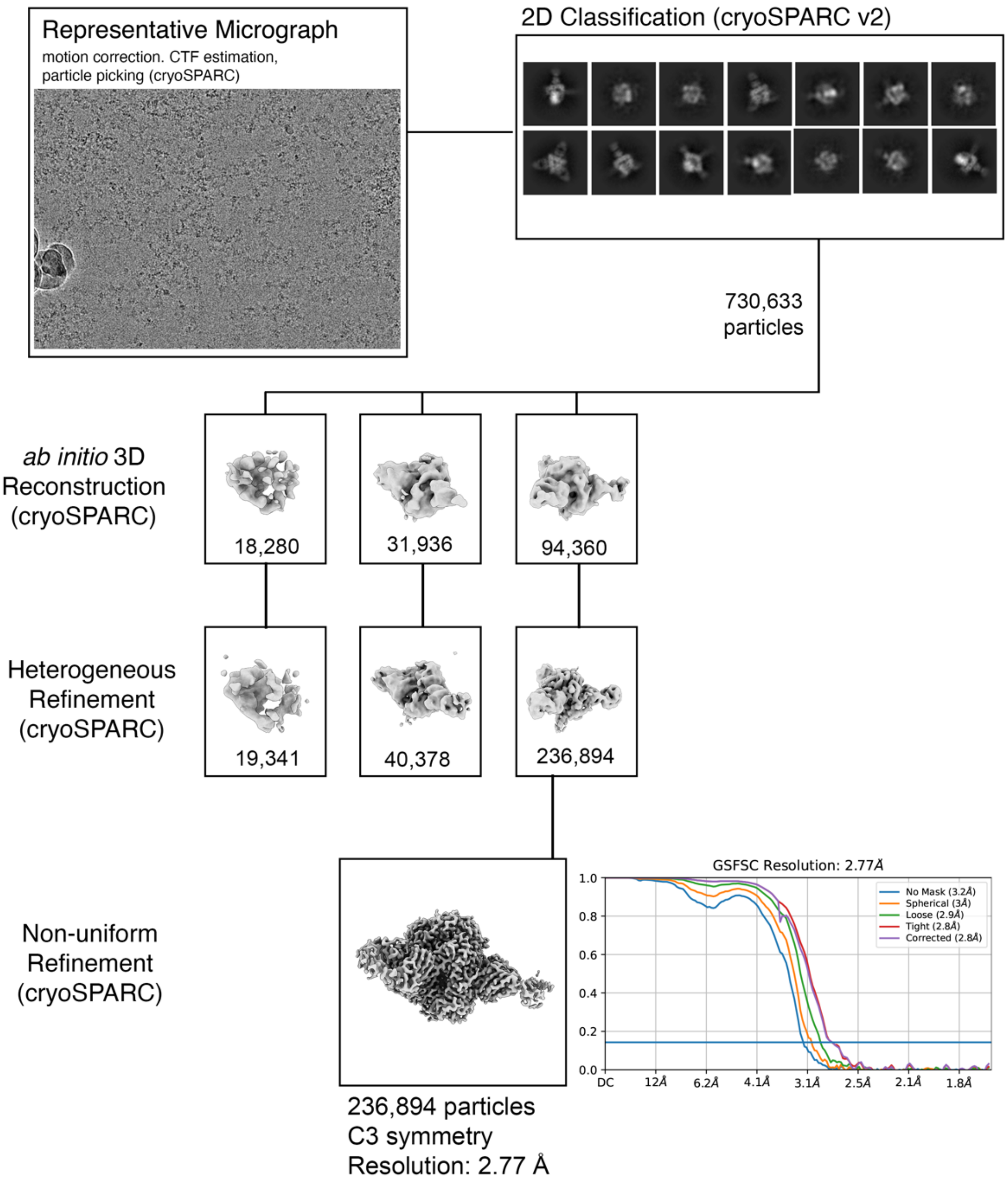

Supplementary Figures 3-8. Cryo-EM processing. Prefusion NiV F alone and in complex with Fabs 4H3, 2D3, 1H8, 1A9, 1H1, 2B12. Each supplementary figure shows a representative motion-corrected micrograph, 2D class averages, and volumes from 3D reconstruction and refinement. Particle numbers and software programs are listed beside each step. High-resolution 3D reconstructions are shown at the bottom of each page, along with their symmetry and gold standard Fourier shell correlation resolutions.

Supplementary Figure 7. Cryo-EM Processing: Prefusion NiV F in complex with Fab 1H1

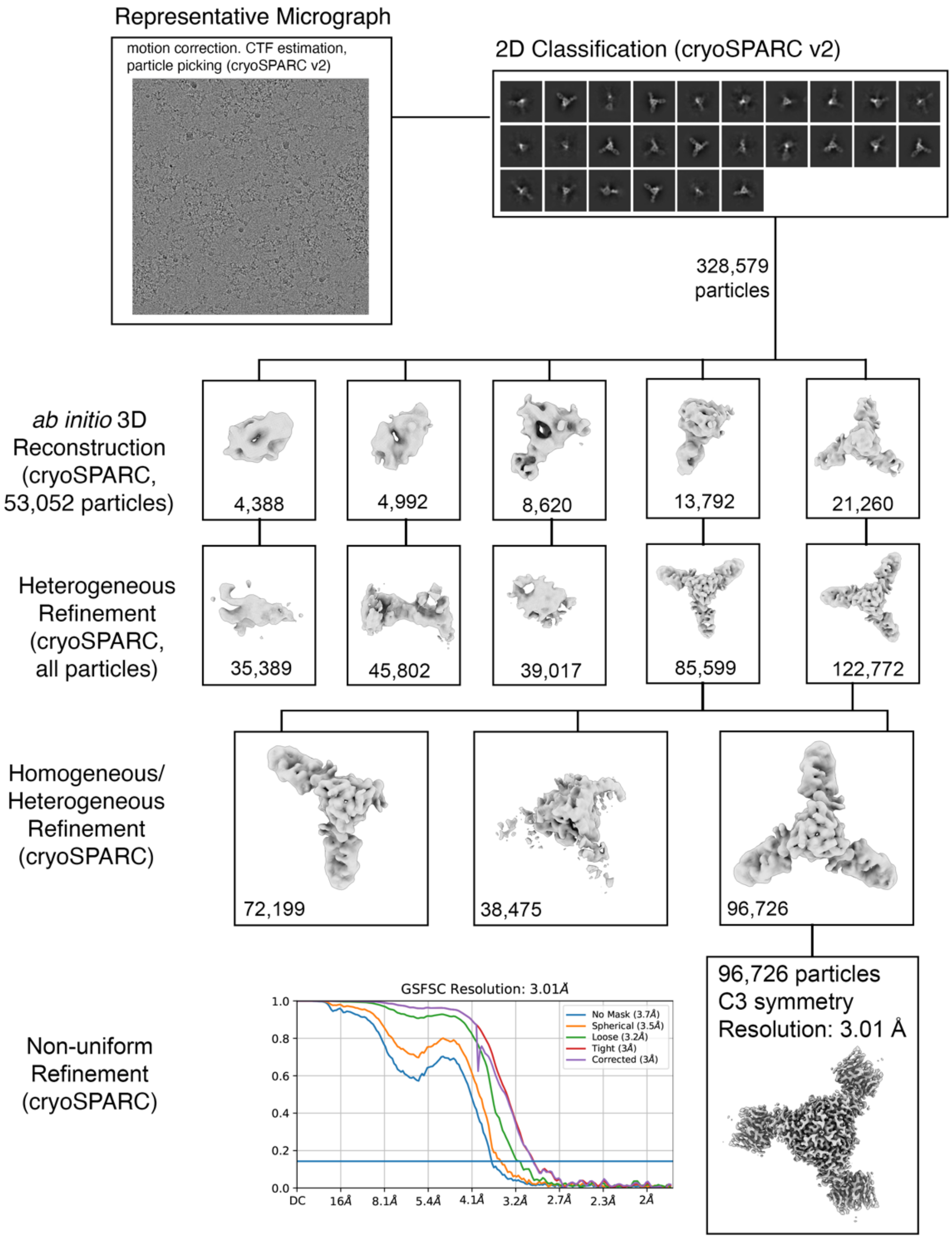

Supplementary Figures 3-8. Cryo-EM processing. Prefusion NiV F alone and in complex with Fabs 4H3, 2D3, 1H8, 1A9, 1H1, 2B12. Each supplementary figure shows a representative motion-corrected micrograph, 2D class averages, and volumes from 3D reconstruction and refinement. Particle numbers and software programs are listed beside each step. High-resolution 3D reconstructions are shown at the bottom of each page, along with their symmetry and gold standard Fourier shell correlation resolutions.

Supplementary Figure 8. Cryo-EM Processing: Prefusion NiV F in complex with Fab 2B12

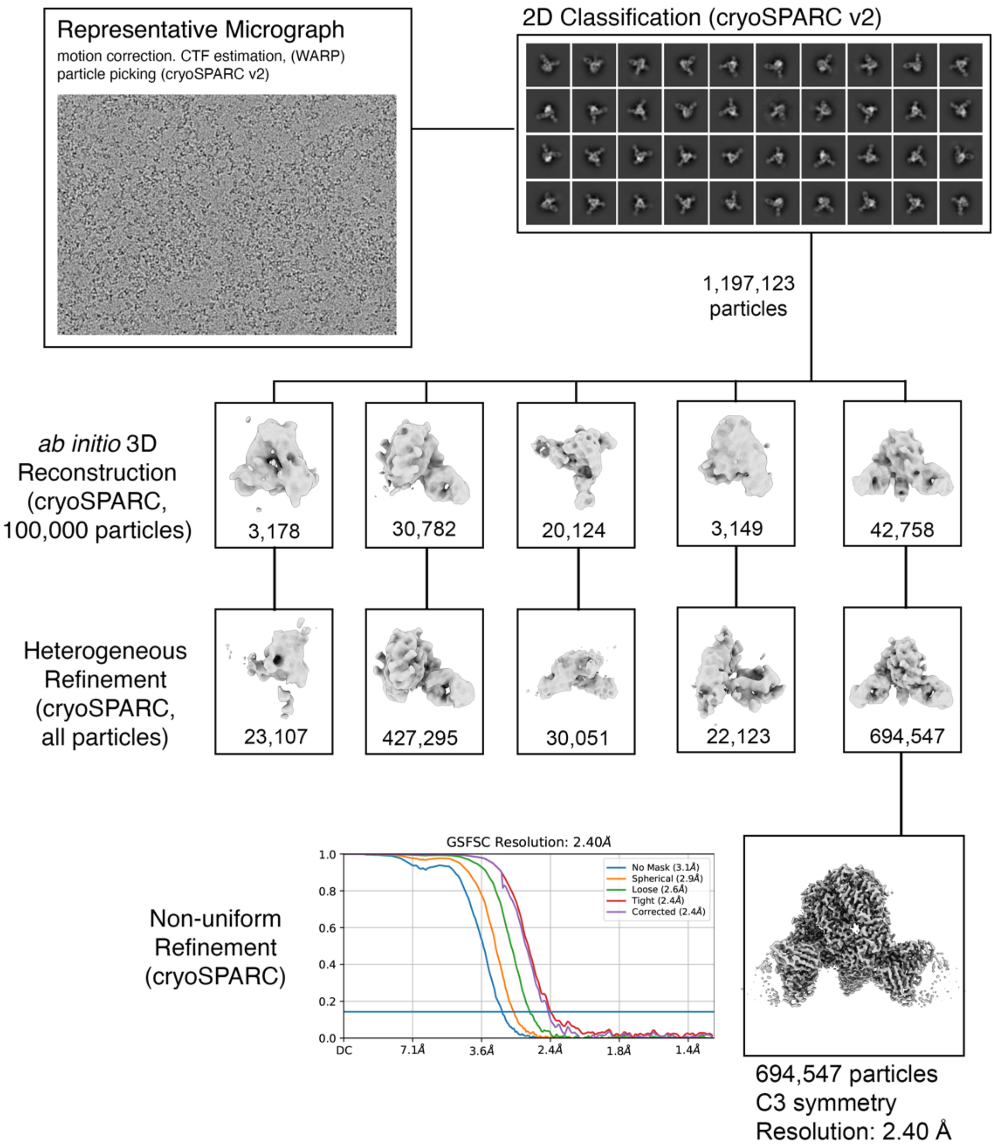

Supplementary Figures 3-8. Cryo-EM processing. Prefusion NiV F alone and in complex with Fabs 4H3, 2D3, 1H8, 1A9, 1H1, 2B12. Each supplementary figure shows a representative motion-corrected micrograph, 2D class averages, and volumes from 3D reconstruction and refinement. Particle numbers and software programs are listed beside each step. High-resolution 3D reconstructions are shown at the bottom of each page, along with their symmetry and gold standard Fourier shell correlation resolutions.

Supplementary Figure 9. Local Resolution Estimates for 3D Reconstructions of prefusion NiV F in complex with Fabs

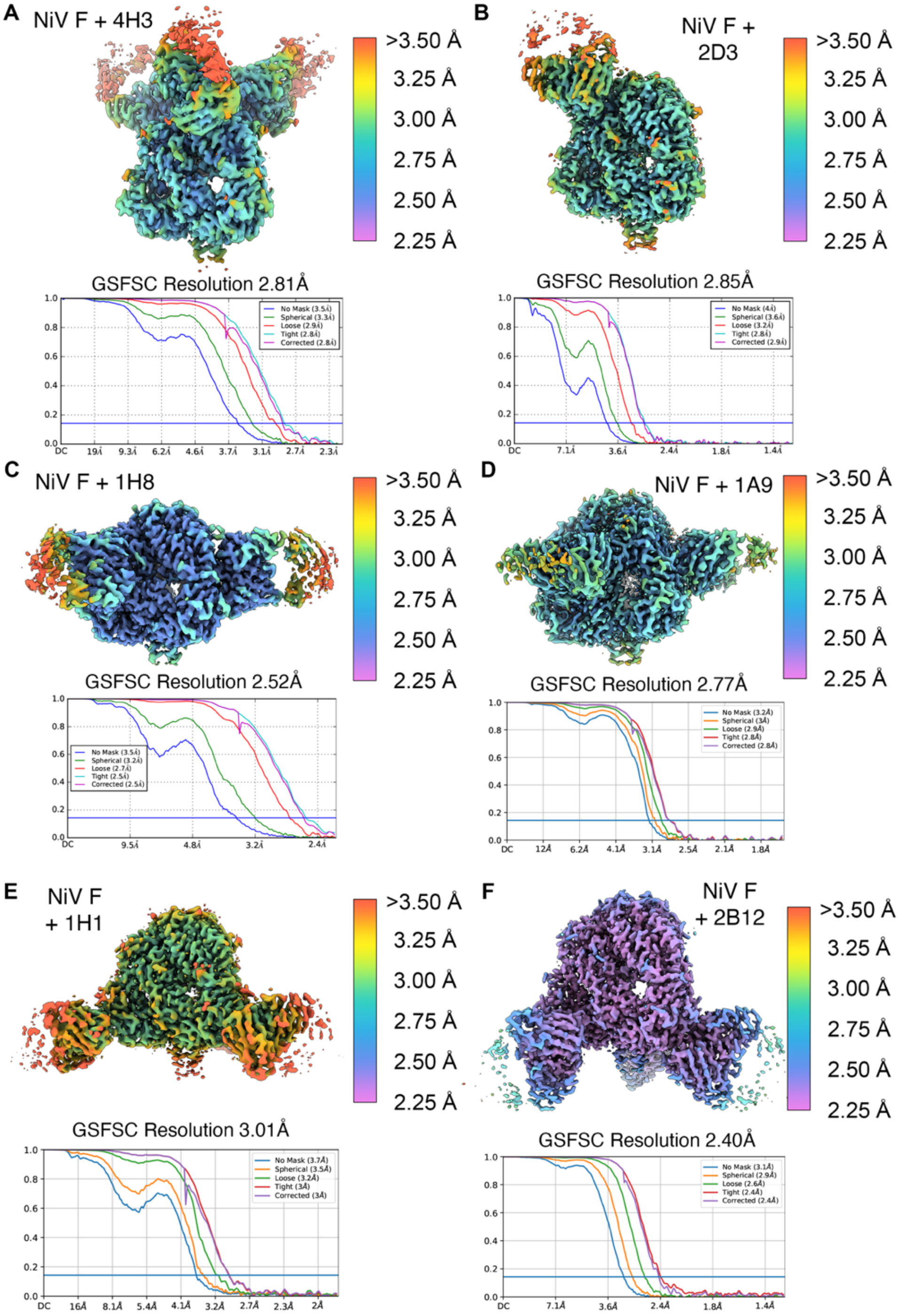

Supplementary Figure 9. Local Resolution Estimates for 3D Reconstructions of prefusion NiV F in complex with Fabs. (A-H) Three-dimensional reconstructions of NiV F complexed with Fabs, colored by the estimated the local resolution. Antibodies are labeled next to each reconstruction. Colored resolution scales are shown below each panel. The same scale (2.25 Å to >3.5 Å) is used for every reconstruction. Estimates were calculated in cryoSPARC.

Supplementary Figure 10. Medium-resolution mapping of additional antibody epitopes on NiV F

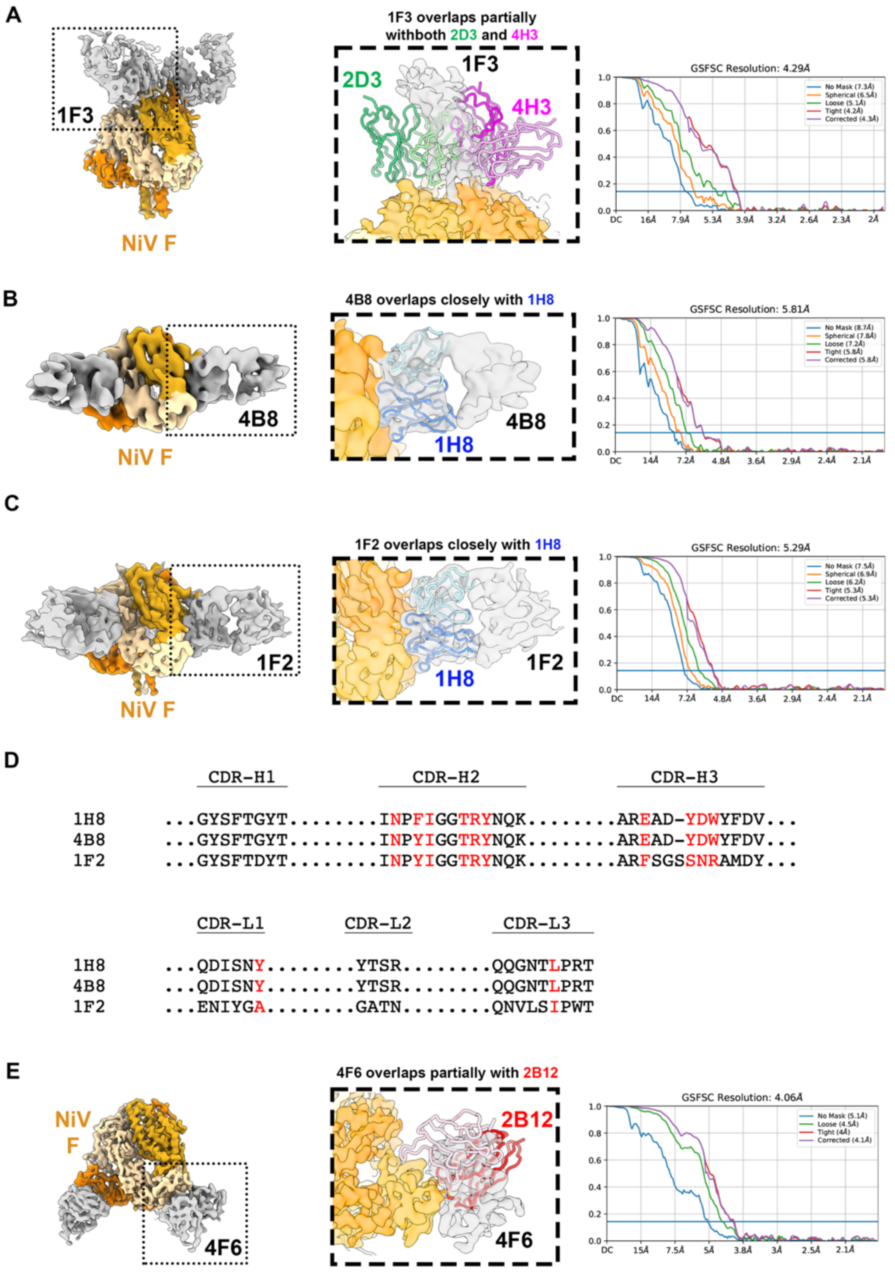

Supplementary Figure 10. Medium-resolution mapping of additional antibody epitopes on NiV F. Each panel shows a different 3D cryo-EM reconstruction of an antibody Fab in complex with prefusion NiV F. Antibodies 1F3, 4B8, 1F2 and 4F6 are shown in panels A, B, C and E, respectively. (D) Multiple sequence alignment of 1H8, 4B8, 1F2. NiV F protomers are colored orange, tan and yellow. Fabs are colored gray. Zoomed insets show overlays of the 3D reconstruction with high resolution Fab models from Figures 4-5. Gold standard Fourier shell correlation resolution plots are shown at the right of each panel.

Supplementary Figure 11. Multiple sequence alignment of henipavirus F proteins

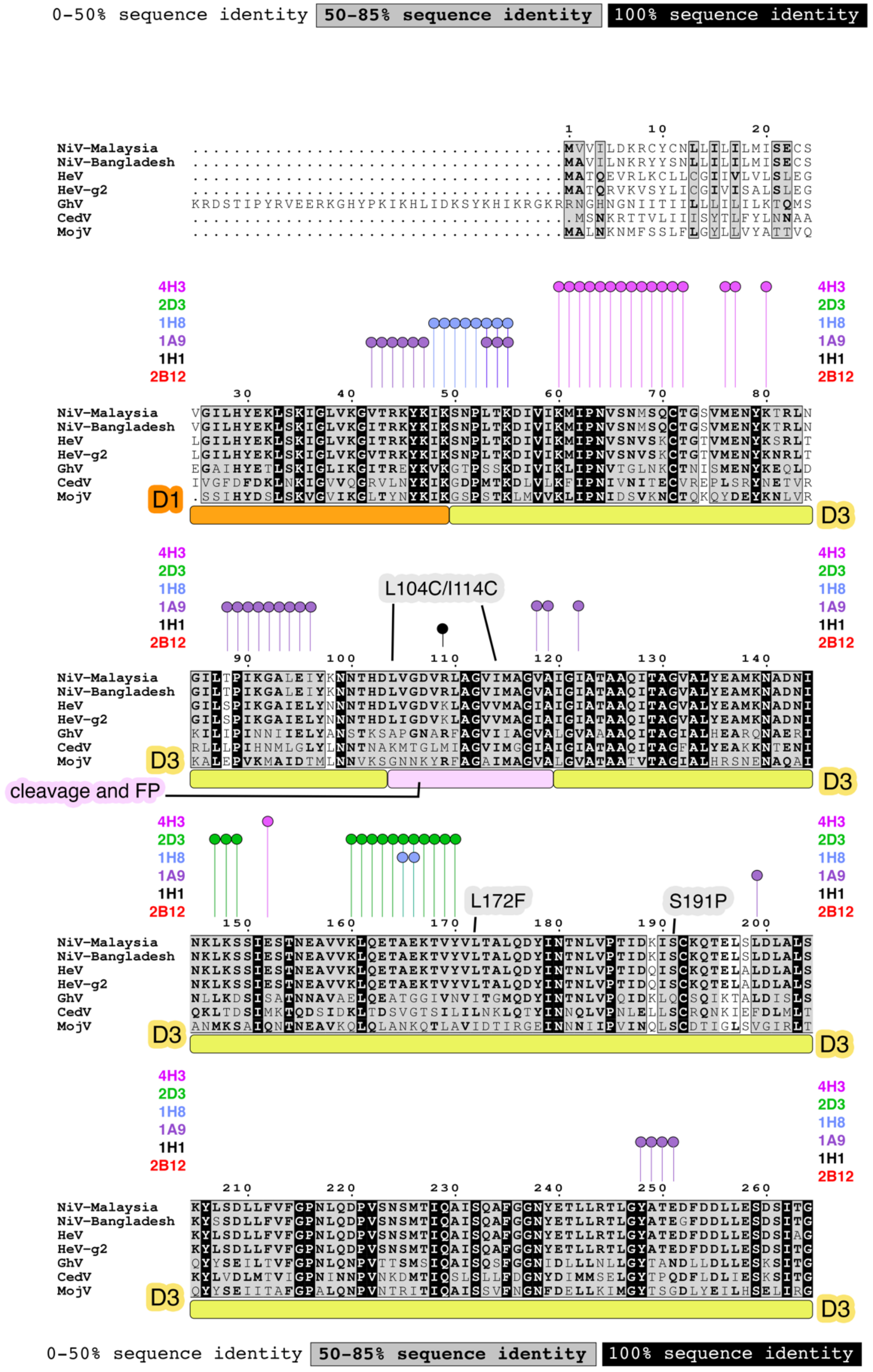

Supplementary Figure 11 (cont'd). Multiple sequence alignment of henipavirus F proteins

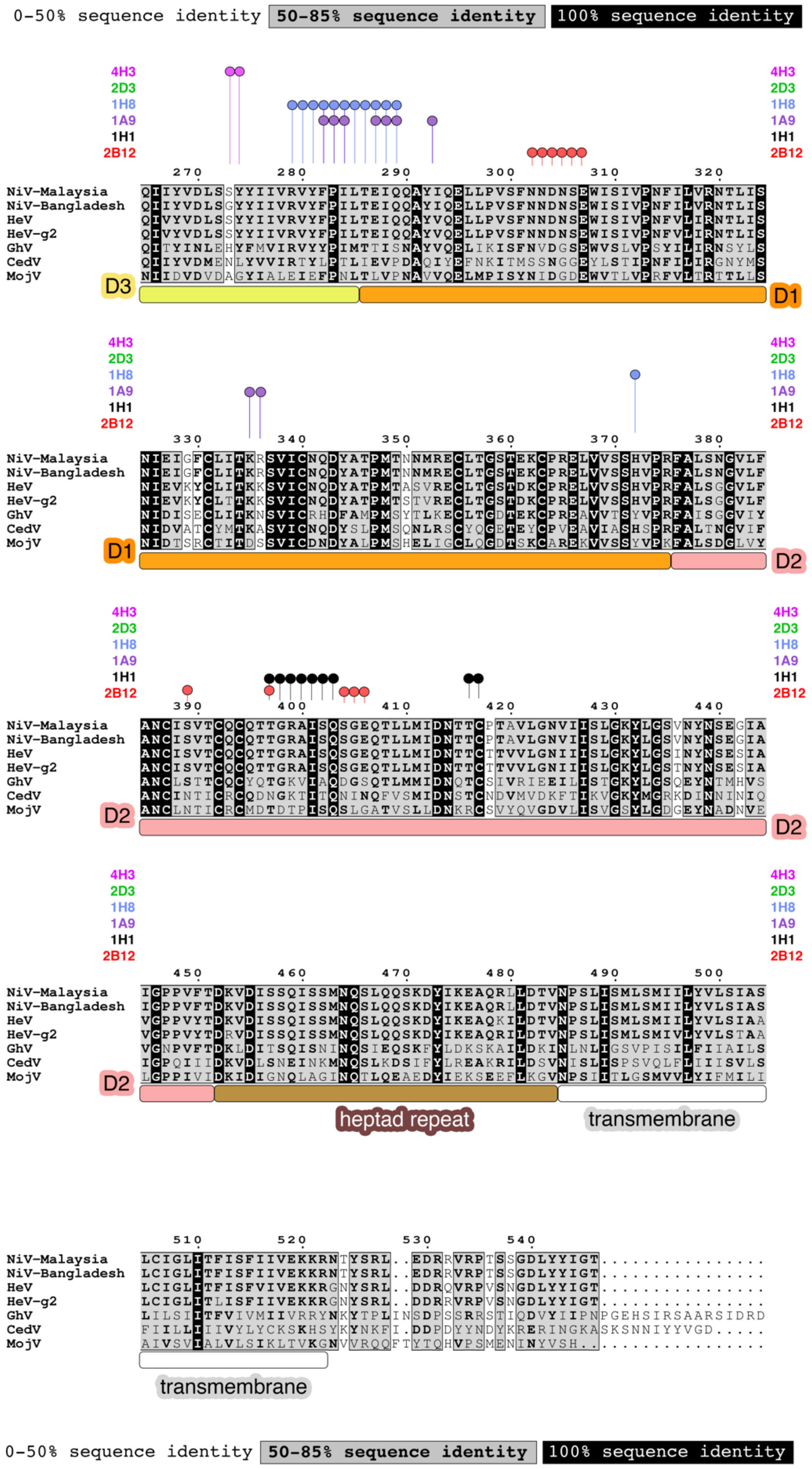

Supplementary Figure 11. Multiple sequence alignment of henipavirus F proteins. Alignment output from Clustal Omega for the F proteins from Nipah henipavirus (NiV-Malaysia AAK29087.1), Nipah henipavirus (NiV-Bangladesh AAY43915.1), Hendra henipavirus (HeV NP_047111.2), Hendra henipavirus g2 (HeV-g2 UCY33687.1), Mòjiāng henipavirus (MojV YP_009094094.1), Cedar henipavirus (CedV YP_009094085.1) and Ghanaian bat henipavirus (GhV YP_009091837.1). The six antibodies with high-resolution structural information (4H3, 1D3, 1H8, 1A9, 1H1 and 2B12) are listed above each sequence block, with colorful balls and sticks denoting regions of interaction with an antibody. Black background with white lettering denotes 100% conservation, gray background with black lettering denotes 50-85% conservation, plan black lettering denotes <50% conservation (see coloring legend at top and bottom of each page). Bold lettering indicates sequence identity or strong conservation among sequences.

Supplementary Figure 12. Antibodies elicited by NiV F also bind HeV F

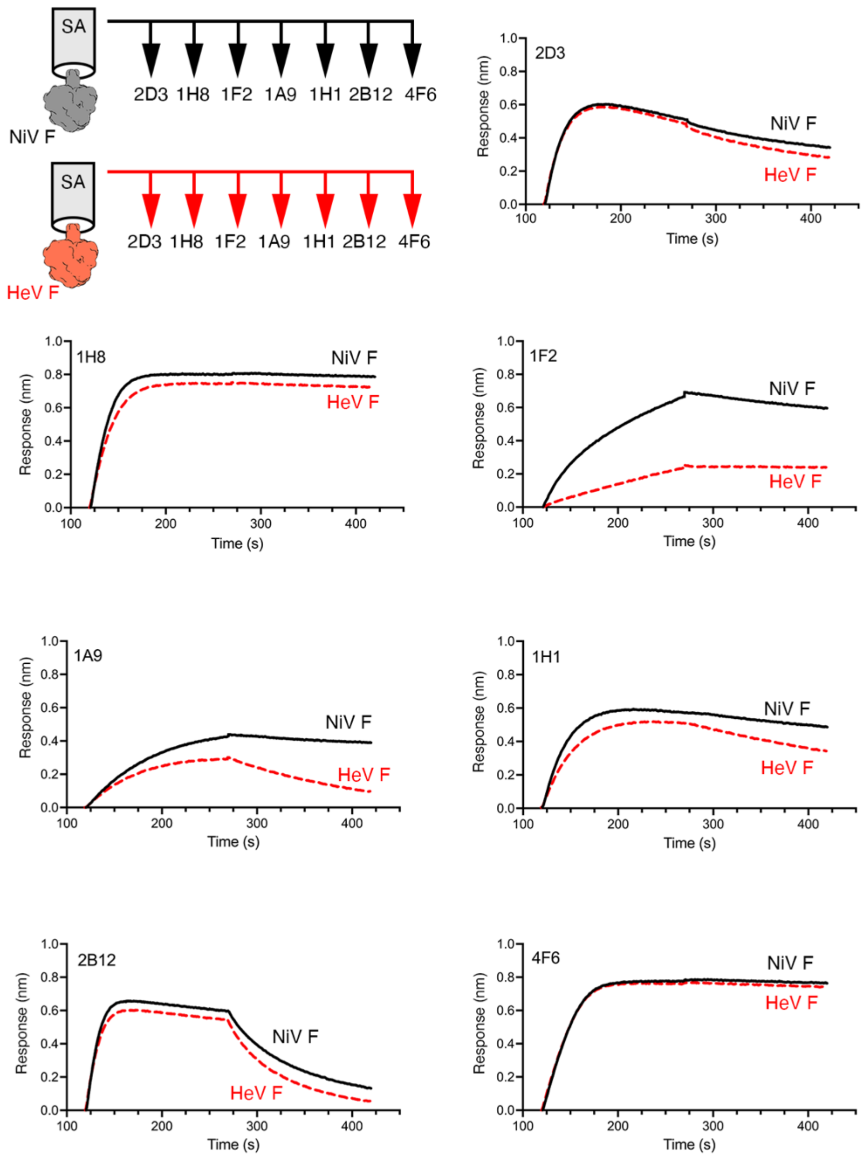

Supplementary Figure 12. Antibodies elicited by NiV F also bind HeV F. Binding of antibodies to prefusion NiV F and prefusion HeV F measured by biolayer interferometry. Streptavidin coated biosensor tips were functionalized with either NiV F or HeV F, then dipped into a panel of antibodies directed against the apical, lateral, and basal faces of the F protein. Curves show the baseline-corrected response units in nanometers as a function of time in seconds. NiV F curves are shown in solid black, HeV curves are shown in dashed red. Antibodies are indicated at the top left corner of each graph. Cartoons of NiV F and HeV were generated in ChimeraX by simulating cryo-EM maps at low resolution, using the NiV trimers from this paper as a starting model.
