# Supplementary material for: Structural basis for antibody recognition of vulnerable epitopes on Nipah virus F protein
Source: Nat Commun. 2023 Mar 17;14:1494. doi: 10.1038/s41467-023-36995-y (PMC10021056; doi:10.1038/s41467-023-36995-y)
Supplement: Supplementary file 3 — Reporting Summary [file 41467_2023_36995_MOESM3_ESM.pdf]

nature portfolio

Corresponding author(s): Loomis, Rebecca J.

Last updated by author(s): Feb 24, 2023

Reporting Summary

Nature Portfolio wishes to improve the reproducibility of the work that we publish. This form provides structure for consistency and transparency in reporting. For further information on Nature Portfolio policies, see our Editorial Policies and the Editorial Policy Checklist.

Statistics

For all statistical analyses, confirm that the following items are present in the figure legend, table legend, main text, or Methods section.

n/a	Confirmed	
[ ]	[x]	The exact sample size (*n*) for each experimental group/condition, given as a discrete number and unit of measurement
[ ]	[x]	A statement on whether measurements were taken from distinct samples or whether the same sample was measured repeatedly
[x]	[ ]	The statistical test(s) used AND whether they are one- or two-sided<br>*Only common tests should be described solely by name; describe more complex techniques in the Methods section.*
[x]	[ ]	A description of all covariates tested
[ ]	[x]	A description of any assumptions or corrections, such as tests of normality and adjustment for multiple comparisons
[ ]	[x]	A full description of the statistical parameters including central tendency (e.g. means) or other basic estimates (e.g. regression coefficient) AND variation (e.g. standard deviation) or associated estimates of uncertainty (e.g. confidence intervals)
[x]	[ ]	For null hypothesis testing, the test statistic (e.g. *F*, *t*, *r*) with confidence intervals, effect sizes, degrees of freedom and *P* value noted<br>*Give P values as exact values whenever suitable.*
[x]	[ ]	For Bayesian analysis, information on the choice of priors and Markov chain Monte Carlo settings
[x]	[ ]	For hierarchical and complex designs, identification of the appropriate level for tests and full reporting of outcomes
[x]	[ ]	Estimates of effect sizes (e.g. Cohen's *d*, Pearson's *r*), indicating how they were calculated

Our web collection on statistics for biologists contains articles on many of the points above.

Software and code

Policy information about availability of computer code

Data collection	Negative-Stain EM: Serial EM on FEI Tecnai T20 microscope equipped with a 2k x 2k Eagle CCD camera and operated at 200 kV, FACS: FACS Aria II (BD Bioscicences) interfaced with FacsDiva software version 8.0.1 (BD Biosciences), Neutralization assay: SpectraMax L luminometer (Molecular Devices), Biolayer Interferometry: forteBio Octet HTC instrument, Octet Data Acquistion v12.0.2.11, CryoEM: Talos F200C (FEI) transmission electron microscope – to screen for quality control, high-resolution images collected on Titan Krios TEM (ThermoFisher Scientific) operating at 300 kV equipped with a K3 camera (Gatan), 1H1 was imaged on Glacios TEM equipped with a Falcon 4 detector (ThermoFisher Scientific), SPR: Biacore X100 (GE Healthcare)

Data analysis	Negative-stain EM: Manual correction using EMANS, reference-free 2D classifications were performed with Relion 1.4, FACS: FlowJo software version 9.9.4 (Tree Star, Inc), Neutralization assay: IC80 is calculated by curve fitting and nonlinear regression (Log(agonist) vs normal response (variable slope) EC) using GraphPad Prism v8,Biolayer interferometry: Octet Data Analysis v12.0.2.3, Percent competition (PC) of analyte mAbs binding to competitor-bound NiV prefusion F was determined using the equation : PC = 100 – [(analyte mAb binding in the presence of competitor mAb) / (analyte mAb binding in the absence of competitor mAb)] x 100, CryoEM: Movies collected using SerialEM, Motion correction and CTF-estimation performed in WARP or cryoSPARC Live, Micrographs imported into cryoSPARC for particle picking, 2D classification, ab initio 3D reconstruction and 3D refinement, Homology models for Fabs were generated using ABodyBuilder, Initial models were docked into the cryo-EM maps using Chimera, Complementarity-determining loops were built manually in Coot, Models were iteratively refined using Coot, Phenix and ISOLDE, SPR: Data were double reference-subtracted and fit to a 1:1 binding model using Biacore Evaluation Software

For manuscripts utilizing custom algorithms or software that are central to the research but not yet described in published literature, software must be made available to editors and reviewers. We strongly encourage code deposition in a community repository (e.g. GitHub). See the Nature Portfolio guidelines for submitting code & software for further information.

Data

Policy information about availability of data

All manuscripts must include a data availability statement. This statement should provide the following information, where applicable:

- Accession codes, unique identifiers, or web links for publicly available datasets
- A description of any restrictions on data availability
- For clinical datasets or third party data, please ensure that the statement adheres to our policy

Structural models are deposited in the protein data bank (PDB, https://www.rcsb.org/) and are scheduled to be released upon publication of this paper. The PDB IDs are: 7UOP, 7UP9, 7UPA, 7UPK, 7UPB, 7UPD. Cryo-EM maps are deposited in the EM Database (https://www.emdataresource.org/) and are scheduled to be released upon publication of this paper. The EMD IDs are: 26652, 26658, 26659, 26668, 26660, 26662. All data supporting the findings of this study are within the article, its Supplementary Information files or provided as a Source Data file.

Human research participants

Policy information about studies involving human research participants and Sex and Gender in Research.

Reporting on sex and gender	NA
Population characteristics	NA
Recruitment	NA
Ethics oversight	NA

Note that full information on the approval of the study protocol must also be provided in the manuscript.

Field-specific reporting

Please select the one below that is the best fit for your research. If you are not sure, read the appropriate sections before making your selection.

☒ Life sciences ☐ Behavioural & social sciences ☐ Ecological, evolutionary & environmental sciences

For a reference copy of the document with all sections, see nature.com/documents/nr-reporting-summary-flat.pdf

Life sciences study design

All studies must disclose on these points even when the disclosure is negative.

Sample size	10 female CB6F1/J mice were immunized, spleens from 4 randomly selected mice were pooled for B-cell sorting/antibody isolation.
Data exclusions	No data was excluded.
Replication	Initial screening/characterization of identified antibodies included binding analysis, neutralization assays and competition binding studies. All studies or relevant portions of studies (i.e. competition binding studies with identified neutralizing antibodies competing with other neutralizing antibodies) were repeated with comparable results at least twice.
Randomization	Animals were randomly allocated to immunization groups at start of study.
Blinding	No blinding to immunization was used. This is a non-clinical study with data collection and analyses relying on objective measures.

Reporting for specific materials, systems and methods

We require information from authors about some types of materials, experimental systems and methods used in many studies. Here, indicate whether each material, system or method listed is relevant to your study. If you are not sure if a list item applies to your research, read the appropriate section before selecting a response.

Materials & experimental systems

n/a	Involved in the study
☐	☒ Antibodies
☐	☒ Eukaryotic cell lines
☒	☐ Palaeontology and archaeology
☐	☒ Animals and other organisms
☒	☐ Clinical data
☒	☐ Dual use research of concern

Methods

n/a	Involved in the study
☒	☐ ChIP-seq
☐	☒ Flow cytometry
☒	☐ MRI-based neuroimaging

Antibodies

Antibodies used	anti-VSV G 8G5 monoclonal antibody (Kerafast, clone 8G5F11, catalog #EB0010), 5B3 Nipah F specific monoclonal antibody - we expressed and purified<br>the antibody using published sequence),antibodies for B-cell sorting panel were anti-B220-BV421 (Biolegend, #103240), anti-F4/80-BV510 (Biolegend,<br>#123135), anti-Gr-1-BV510 (Biolegend, #108438), anti-CD4-BV510 (Biolegend, #100449), anti-CD8-BV510 (Biolegend, #100752), anti-IgG(1/2/3)-FITC<br>(BD Pharmingen, #553443, #553399, #553403), anti-IgM-PE-Cy7 (Southern Biotech, #1140-17), monoclonal antibodies described in this manuscript were<br>generated from B-cell sorting, sequenced, cloned, expressed and purified as described in manuscript.
Validation	VSV G antibody was validated in infection assays of VSVΔG-G-luc stock and NiV F/G VSVΔG-luc stock preparations, 5B3 antibody was validated<br>by binding assays (to pre-F and post-F designs), pseudovirus neutralization assays and negative stain EM bound to Nipah F protein, antibody panel<br>used for B-cell sorting were titrated using FACS as described in the methods and shown in Supplementary Figure 1 with final dilution for sort selected highlighted in Supplementary Figure 1B.

Eukaryotic cell lines

Policy information about cell lines and Sex and Gender in Research

Cell line source(s)	Vero E6 cells were purchased from ATCC (VERO C1008, clone E6, catalog number - CRL-1586)
Authentication	Cells lines were not authenticated. They were purchased directly from vendor and maintained and frozen according to manufacturer's instructions.
Mycoplasma contamination	Cell lines were not tested for mycoplasma contamination.
Commonly misidentified lines<br>(See ICLAC register)	Cell lines were not tested for mycoplasma contamination.

Animals and other research organisms

Policy information about studies involving animals; ARRIVE guidelines recommended for reporting animal research, and Sex and Gender in Research

Laboratory animals	CB6F1/J mice from Jackson Laboratory, female, all mice were 6-8 weeks old at start of vaccination. Mice were maintained at 72°F +/-5°F,<br>relative humidity of 30-70% (typically 33-40%) on a 12h light/dark cycle with food and water ad libitum.
Wild animals	No wild animals were used in this study.
Reporting on sex	Only female mice were used in this study. Male mice are more aggressive than female mice. Subsequent studies in ferrets showed no variability in immunogenicity between the sexes.

Field-collected samples	No field-collected samples were used in this study.
Ethics oversight	All animal experiments were reviewed and approved by the Animal Care and Use Committee of the Vaccine Research Center, NIAID, NIH<br>(ACUC animal study protocol #VRC-17-709) and all animals were housed and cared for in accordance with local, state, federal and institute<br>policies in an American Association for Accreditation of Laboratory Animal Care (AAALAC)-accredited facility at the NIH.

Note that full information on the approval of the study protocol must also be provided in the manuscript.

Flow Cytometry

Plots

Confirm that:

- [x] The axis labels state the marker and fluorochrome used (e.g. CD4-FITC).
- [x] The axis scales are clearly visible. Include numbers along axes only for bottom left plot of group (a 'group' is an analysis of identical markers).
- [x] All plots are contour plots with outliers or pseudocolor plots.
- [x] A numerical value for number of cells or percentage (with statistics) is provided.

Methodology

Sample preparation	Four CB6F1/J mouse spleens harvested at wk 44, homogenized using gentleMACS SPLEEN_01 program, splenocytes isolated via ACK Lysis Buffer and centrifugation. Splenocytes were stained with a B cell sorting antibody panel and conjugated probe prior to FACS. Refer to methods for more specific details.
Instrument	Four CB6F1/J mouse spleens harvested at wk 44, homogenized using gentleMACS SPLEEN_01 program, splenocytes isolated via ACK Lysis Buffer and centrifugation. Splenocytes were stained with a B cell sorting antibody panel and conjugated probe prior to FACS. Refer to methods for more specific details.
Software	FacsDiva software version 8.0.1 (BD BioSciences) , Analysis performed using Flow Jo software version 9.9.4, (Tree Star, Inc.)
Cell population abundance	Splenocytes from naive animals were processed and stained at the same time as the splenocytes from animals which had been immunized with our desired antigen. We used the gates determined with the naive splenocytes for our sample. NiV prefusion F+ cells represented about 0.583% of IgG+ cells.
Gating strategy	Splenocytes from naive animals were processed and stained at the same time as the splenocytes from animals which had been immunized with our desired antigen. We used the gates determined with the naive splenocytes for our sample. NiV prefusion F+ cells represented about 0.583% of IgG+ cells.

- [x] Tick this box to confirm that a figure exemplifying the gating strategy is provided in the Supplementary Information.
